# Urethral cavernous hemangioma in a female patient: a rare entity

**DOI:** 10.11604/pamj.2015.22.352.8418

**Published:** 2015-12-11

**Authors:** Mustafa Suat Bolat, Kubilay Yüzüncü, Ekrem Akdeniz, Ayse Nurten Demirdoven

**Affiliations:** 1 Samsun Teaching and Education Hospital, Department of Urology, Samsun, Turkey; 2 Private Atasam Hospital, Clinic of Urology, Samsun, Turkey

**Keywords:** Urethral cavernous hemangioma, urinary system, genital infection

## Abstract

Genitourinary hemangiomas are rare entities of the urinary system. We reported a female patient who suffered dyspareunia and intermitant hematuria that was proved as urethral cavernous hemangioma. Despite its benign nature, hemangiomas may recur due to incomplet excision.

## Introduction

Genitourinary hemangiomas are rare entities that may seldom affect lower portion of the urinary system. Urethral hemangiomas are mostly reported men. Female urethra is rarely affected [2, 3]. In this presentation we reported a cavernous hemangioma of the urethra in a female patient who suffered dyspareunia and intermitant hematuria.

## Patient and observation

**Figure 1:**
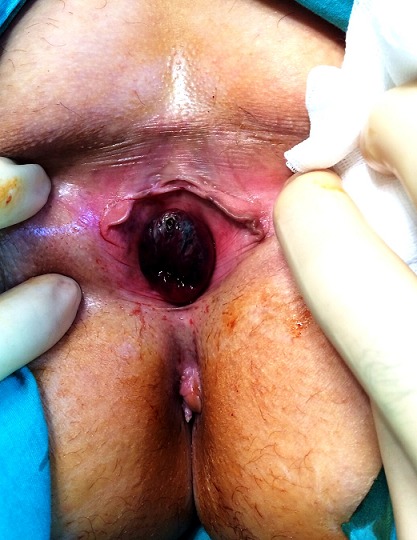
Preoperative appearence of urethral hemangioma

**Figure 2:**
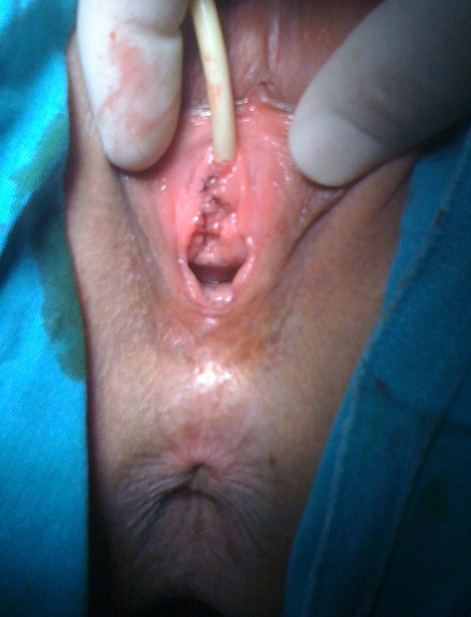
Postoperative appearance

**Figure 3:**
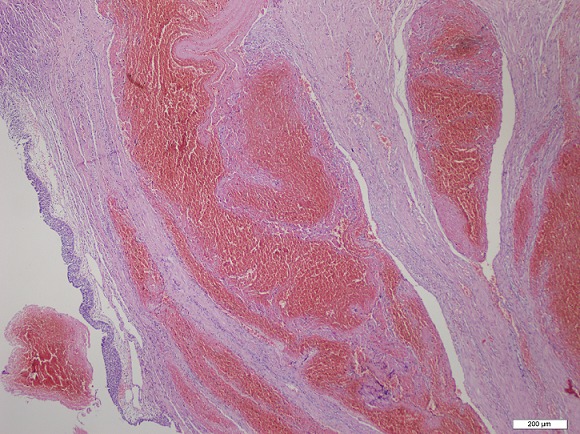
Encapsulated mass was composed of large, cavernous vascular spaces filled with blood cells

Fifty one year old female patient admitted to our clinic with difficulty of urination, dyspareunia and intermitant hematuria. Her medical history revealed no genital surgery and genital infection. On her physical examination, there was a painless, reddish lesion in 30 mm diameter at distal part of urethra surrounding external meatus (Figure 1). Laboratory findings were within normal range. On Computerized Tomography (CT) there was no pathological finding in upper part of the urinary system. After confirming the cystoscopy was normal, urethral mass excision was planned. A foley catheter was placed and mass was excised completely. External meatus was everted with interrupted 3-0 synthetic absorbable sutures (Figure 2). Pathological examination revealed an encapsulated mass which was composed of large, cavernous vascular spaces filled with blood and separated by connective tissue stroma diagnosed as cavernous hemangioma of the urethra (Figure 3). Foley catheter was withdrawn five days after the procedure. The patient was symptom free at seven month follow-up with no evidence of recurrence.

## Discussion

Hemangioma is a very rare pathology and it may be seen in the kidney, ureter, bladder, prostate and urethra [1]. In radiological evaluation, there was no pathological findings in upper urinary system. Involvement of the urethra is extremely rare in women, and reports have been presented as only case reports [2, 3]. The most common symptom is hematuria but patients may also present with urethral mass. In concordance with literature, our patient suffered from intermitant hematuria and frequency on urination. Differential diagnosis of urethral hemangiomas should be considered with malign and benign conditions such as carcinomas, caruncula and periurethral abscess. In spite of benign nature, hemangiomas may recur due to incomplet excision. Other theurapotic modalities are electrocautery or laser ablation [4].

## Conclusion

Differential diagnosis of urethral hemangiomas should be considered with malign and benign conditions such as carcinomas, caruncula and periurethral abscess. In spite of benign nature, hemangiomas may recur due to incomplet excision.
